# Delirium Tremens in a Child

**Published:** 1854-09

**Authors:** 


Delirium Tremens in a Child.—The *Winchester Emblem*, Iowa, mentions a fatal case of the Delirium Tremens in a child four years of age. The boy was a common *drunkard*. The *Emblem* says, that on the 18th ult., his father, who had been fishing, gave the child a bottle of whiskey to carry; he drank too much and was taken very sick, then with twitching in one arm and side, which was soon followed by the delirium tremens which lasted twelve hours.

It was a terrible sight to see the little fellow screaming at and jumping from the snakes he thought he saw.

